# Dynamics of *Legionella* Community Interactions in Response to Temperature and Disinfection Treatment: 7 Years of Investigation

**DOI:** 10.1007/s00248-021-01778-9

**Published:** 2021-06-05

**Authors:** Luna Girolamini, Silvano Salaris, Maria Rosaria Pascale, Marta Mazzotta, Sandra Cristino

**Affiliations:** Department of Biological, Geological, and Environmental Sciences, University of Bologna, via San Giacomo 12, 40126 Bologna, BO Italy

**Keywords:** *Legionella* community, *Legionella* interaction, Resistance, *L. anisa*, *L. rubrilucens*, *L. pneumophila*

## Abstract

In man-made water distribution systems, *Legionella* community interactions remain unknown, due to their ability to change from sessile to planktonic states or live in viable but non-culturable forms, in response to anthropic and environmental stress. During 7 years of hospital *Legionella* surveillance, in 191 hot water positive samples, the interactions among the *Legionella* species, temperature, and disinfection treatment were evaluated. *Legionella* was isolated following ISO 11731:2017, and identification was performed by *mip* gene sequencing and sequence-based typing (SBT) for *L. anisa* or *L. rubrilucens* and *L. pneumophila*, respectively. The species with the higher frequency of isolation was *L. pneumophila* serogroup 1 (78.53%; 4865.36 ± 25,479.11 cfu/L), followed by *L. anisa* (54.45%; 558.79 ± 2637.41 cfu/L) and *L. rubrilucens* (21.99%; 307.73 ± 1574.95 cfu/L), which were sometimes present together. Spearman’s rho correlation test was conducted among the species with respect to temperature and disinfectant (H_2_O_2_/Ag^+^). The results showed a generally positive interaction among these species sharing the same environment, except for competition between *L. anisa* and *L. rubrilucens*. High temperature (48.83 ± 2.59 °C) and disinfection treatment (11.58 ± 4.99 mg/L) affected the presence of these species. An exception was observed with *L. anisa*, which showed disinfection treatment resistance. For the purposes of environmental surveillance, it is fundamental to better understand the interactions and dynamic of the *Legionella* community in man-made water systems in order to choose the proper physical or chemical treatments. The simultaneous presence of different *Legionella* species could result in an increased resistance to high temperature and disinfectant treatment, leading to changes in contamination level and species diversity.

## Introduction

*Legionella* species are Gram-negative and ubiquitous bacteria that are inhabitants of fresh and artificial water systems and biofilms. The *Legionella* genus includes up to 60 species [1] and more than 70 different serogroups [2]. Approximately 20 species of *Legionella* have been proven to be causative agents of Legionnaires’ disease (LD). In total, 85% of human diseases are caused by *L. pneumophila* (*Lp*) serogroup 1 (*Lp*1) [3]. About 10% of human infections are caused by non-*pneumophila Legionella* species (n-*pL*), especially *L. micdadei*, *L. bozemanae*, *L. longbeachae*, *L. dumoffii*, and *L. feeleii*, which are repeatedly isolated from hospitalized patients, whereas *L. anisa*, *L. wadsworthii*, and *L. cincinnatiensis* are only rarely found [4, 5]. The differences described could probably be attributed to the specificity and sensitivity of the antigenic urinary diagnostic test for *Lp*, and especially *Lp*1 [2, 6].

The temperature is one of the most important factors for the presence and growth of *Legionella* in environments and pipeline water distribution systems. *Legionella* can survive for long periods in several natural and artificial environments at fairly high temperatures and in the presence of disinfectants, resisting anthropogenic and environmental stress [7]. *Legionella* are mesophilic bacteria; they can survive in a temperature ranging from 5.7 to 63 °C, but their optimal growth temperature is between 25 and 40 °C [8, 9]. For this reason, to decrease the risk of legionellosis, a high temperature (above 50 °C) is highly recommended [10–12]. In addition, a wide variety of disinfection techniques, including chemical disinfection (chlorine dioxide, monochloramines, and hydrogen peroxide, etc.) and physical treatments like ultraviolet (UV) light and high temperature (shock treatment), have been employed worldwide as strategies to reduce the risk of legionellosis [13, 14]. Due to the ability of *Legionella* to survive in cysts of free-living protozoa, such as several ameba species (e.g., *Acanthamoeba polyphaga*, *A. castellani*, *Vermamoeba vermiformis*, or *Dictyostelium discoideum*), as well as in biofilm aggregates, they can find protection against environmental and anthropic stress (e.g., disinfectants) [15–17]. As previously documented, the disinfection of water distribution systems, as well as changes in environmental conditions (e.g., pH, nutrition levels, and water flow), permits the release of *L. pneumophila* that is able to alternate between a sessile (biofilm) and a free-living planktonic state [18, 19]. Moreover, the corrosion of the biofilm surface permits bacterial dispersion, increasing the risks of transmission by water contact or aerosolization [20, 21]. In man-made water distribution systems, *Lp* and n-*pL* can live together or alone [22–26]. It is poorly known if bacteria belonging to *Legionella* species compete or exhibit commensal interactions in the same environment.

This study aimed to understand the interactions and dynamics of the *Legionella* community in the hot water distribution system of an Italian hospital, during 7 years of *Legionella* environmental surveillance. Based on our previous findings [27, 28], the molecular characterization of *Lp *and n-*Lp *species highlighted the presence of a variegated environment and suggested a need to study the complex community found, in order to obtain detailed insights into the community ecology of *Legionella* species and their responses to temperature and disinfection treatment. Our knowledge of the hospital permitted us to study the interaction between the *Lp* and n-*pL* populations, and their response to chemical and physical treatments.

## Methods

### Hospital Water Safety Plan and Disinfection Treatment

This study was conducted in an Italian hospital which, according to Italian Guidelines [29], had a water safety plan (WSP) involving a *Legionella* surveillance program consisting of hot water distribution system sampling performed four times per year. The characteristics of the risk assessment plan of the hospital have been previously described [27].

Briefly, control of *Legionella* contamination in the hospital started from 2013. The hospital hot water distribution systems were treated with a disinfectant based on a stabilized combination of hydrogen peroxide (H_2_O_2_) (34%, wt/wt) and silver (Ag^+^) salts (0.003%, wt/wt) (H_2_O_2_/Ag^+^).

### Sample Collection

From 2013 to 2019, 307 hot water samples (2 L) were collected in the post-flushing modality [30]. During sampling, values of temperature and disinfectant residues were measured and recorded at distal outlets.

### Microbiological Analysis and Isolate Identification

The isolation of *Legionella* from hot water samples was performed by the culture technique according to the ISO 11731:2017. The fixed detection limit was 50 colony-forming units per liter (cfu/L) [31]. The culture takes a minimum of 10 to up 15 days, and every 2 days, the plates were examined and the presumptive colonies were enumerated and sub-cultured on BCYE agar with *L-cysteine* (*cys*+) and without *L-cysteine *(*cys*-) (Thermo Fisher Scientific, Diagnostic, Ltd., Basingstoke, UK). The *Legionella* colonies’ growth was observed only on BCYE agar with *L-cysteine *(BCYE cys+). These *Legionella* colonies (at least five different colonies for each plate) were identified using the *Legionella* latex test kit (*Legionella* latex test kit; Thermo Fisher Scientific, Ltd. Basingstoke, UK), based on the manufacturer’s instructions.

The data obtained were expressed as the mean concentration ± standard deviation (SD), and colony-forming units/l (cfu/L).

Molecular gold standard techniques for *Legionella* identification were applied [32, 33]. Briefly, Genomic DNA was extracted from isolates by the InstaGene Purification Matrix (Bio-Rad, Hercules, CA). Gene amplification was carried out in a 50-μL reaction volume containing DreamTaq Green PCR Master Mix 2× (Thermo Fisher Scientific) and 40 pmol of each primer, while 100 ng of DNA was used as template. Following purification, DNA was sequenced using BigDye Chemistry and analyzed on an ABI PRISM 3100 Genetic Analyzer (Applied Biosystems, Foster City, CA). All strains identified as *L. pneumophila* by agglutination tests were analyzed by sequence-based typing (SBT) to determine the sequence type (ST) according to an ELDSNet protocol (http://bioinforatics.phe.org.uk/legionella/legionella_sbt/php/sbt_homepage.php). ST allelic profile was assigned by the ELDSNet database (http://www.hpabioinformatics.org.uk/cgibin/legionella/sbt/seq_assemble_legionella1.cgi); strains identified as *Legionella species* were analyzed by *mip* sequencing, as described by Ratcliff et al. [34]. The sequences were compared with sequences deposited in the *Legionella mip* gene database using a similarity analysis tool (http://bioinformatics.phe.org.uk/cgi-bin/legionella/mip/mip_id.cgi). The identification at the species level was conducted based on ≥98% similarity to a sequence in the database [35].

### Statistical Analysis

Statistical analyses were performed using R Statistical Software (version 4.0.2, “Taking Off Again” R Foundation for Statistical Computing, Vienna, Austria). The Shapiro–Wilk test was performed to study the variables’ normality. In order to evaluate whether the samples originated from the same distribution, Mann–Whitney tests were carried out. Spearman’s rho rank correlations were calculated for each pairwise combination (*Lp*1, *L. anisa*, *L. rubrilucens*, temperature, and disinfectant) in nine different groups identified to evaluate the interaction among the species.

Spearman’s rho coefficient was used to classify the correlation found according to Asuero et al. [36], as follows:

- ±0.90 to ±1.00: Very high correlation;
- ±0.70 to ±0.89: High correlation;
- ±0.50 to ±0.69: Moderate correlation;
- ±0.30 to ±0.49: Low correlation;
- 0.00 to ±0.29: Little if any correlation.

The significance of all statistical tests was set at a *p* value of (*p*) ≤ 0.05.

## Results

### Physical and Chemical Parameters

During the study, temperature and disinfectant residues (H_2_O_2_/Ag^+^) were measured for all outlets sampled. The hospital’s mean temperature and disinfectant concentration, measured at distal outlets, were 48.83 ± 2.59 °C and 11.58 ± 4.99 mg/L, respectively. In detail, Table 1 shows the physical–chemical parameters measured.

**Table 1:** Data collected for the nine groups: mean *Legionella* concentration, temperature, and disinfectant residues

Groups	Number of samples	Parameters	*Lp*1 concentration (cfu/L)	*L. anisa* concentration (cfu/L)	*L. rubrilucens* concentration (cfu/L)	Temperature (°C)	H_2_O_2_ (mg/L)
A	191	Mean ± SD	4865.36 ± 25,479.11	558.79 ± 2637.41	307.73 ± 1574.95	48.83 ± 2.59	11.58 ± 4.99
		Min–Max	0–251,400.00	0–29,000.00	0–14,100.00	32.5–54.50	0–25.00
		Median	100.00	12.50	0	49.17	10.60
B	67	Mean ± SD	3854.08 ± 21,660.98			49.28 ± 3.05	10.47 ± 5.35
		Min–Max	8.33–175,250			32.50–54.50	0–21.00
		Median	166.67			49.50	10.00
C	41	Mean ± SD		545.34 ± 946.45	73.65 ± 390.42	48.81 ± 2.84	13.32 ± 5.55
		Min–Max		0–3250.00	0–2500.00	33.27–51.10	1.50–25.00
		Median		50.00	0	49.40	13.33
D	33	Mean ± SD		510.87 ± 874.22		48.71 ± 3.04	14.30 ± 5.21
		Min–Max		7.14–3250.00		33.27–51.10	5.00–25.00
		Median		50.00		49.30	14.00
E	5	Mean ± SD			48.93 ± 33.49	50.20 ± 0.51	9.80 ± 6.68
		Min–Max			7.14–100.00	49.60–50.80	1.50–20.00
		Median			50.00	50.25	10.00
F	3	Mean ± SD		1833.33 ± 1560.72	925.00 ± 1366.79	47.60 ± 2.36	8.33 ± 2.89
		Min–Max		50.00–2950.00	50.00–2500.00	45.60–50.20	5.00–10.00
		Median		2500.00	225.00	47.00	10.00
G	49	Mean ± SD	4881.40 ± 22,454.70	1497.32 ± 5018.80		48.46 ± 2.17	11.83 ± 4.02
		Min–Max	16.67–156,612.50	6.25–29,000.00		37.83–52.02	4.25–22.50
		Median	312.50	100.00		48.45	11.67
H	15	Mean ± SD	17,313.74 ± 64,765.97		2259.57 ± 4645.71	49.08 ± 1.00	9.62 ± 4.05
		Min–Max	11.11–251,400.00		11.11–14,100.00	47.20–50.50	1.00–16.25
		Median	125.00		75.00	49.05	10.00
I	19	Mean ± SD	8004.81 ± 17,781.96	578.99 ± 665.79	1150.73 ± 2097.03	48.02 ± 1.92	12.54 ± 4.22
		Min–Max	16.67–72,092.86	16.70–2750.00	16.67–8400.00	42.50–50.86	7.50–20.00
		Median	400.00	391.67	428.57	48.47	10.33

### *Legionella *Community Characteristics

From 307 hot water samples analyzed for detection and enumeration of *Legionella* spp., only the positive samples (191/307, 62.2%) were considered for this study.

The *Legionella* hospital community was represented by three *Legionella* species: *Lp*1 was isolated in 150/191 samples (78.53%; 4865.36 ± 25,479.11 cfu/L) and n-*pL* in 124/191 samples (21.4%; 866.52 ± 3042.75 cfu/L). In some cases, they were present at the same time, in the same sample.

In particular, regarding the 124 n-*pL* positive samples, 104/124 (83.87%; 558.79 ± 2637.41 cfu/L) belonged to *L. anisa* and 42/124 (33.87%; 307.73 ± 1574.95 cfu/L) to *L. rubrilucens*. In some samples, these species were *simultaneously* present.

### Legionella Community Interactions

A total of 191 *Legionella* positive samples were examined in order to evaluate the species interactions within the samples.

A comparison between the two main communities, represented by *Lp*1 vs n-*pL*, performed by the Mann–Whitney test, returned a significant difference (*p* = 0.011), with *Lp*1 higher than n-*pL* (*p* = 5.67 × 10^−3^).

To study the interaction within the *Legionella* species found, the following groups were defined:

A. All 191 *Legionella* positive samples;
B. Samples (n. 67) contaminated by only *Lp*1;
C. Sample (n. 41) contaminated by only n-*pL* (*L. anisa* and/or *L. rubrilucens*);
D. Samples (n. 33) contaminated by only *L. anisa*;
E. Samples (n. 5) contaminated by only *L. rubrilucens*;
F. Samples (n. 3) contaminated by *L. anisa* and *L. rubrilucens*, simultaneously;
G. Samples (n. 49) contaminated by *Lp*1 and *L. anisa*;
H. Samples (n. 15) contaminated by *Lp*1 and *L. rubrilucens*;
I. Samples (n. 19) contaminated by *Lp*1, *L. anisa*, and *L. rubrilucens*, simultaneously.

The *Legionella* contamination levels, with temperature and disinfectant residues, recorded in the nine groups, are shown in Table 1.

For groups A, C, G, H, and I, the Mann–Whitney test was performed to compare the distribution of *Legionella* isolates belonging to different species. Groups B, D, and E could not be subjected to the Mann–Whitney statistical test because they contained a single species.

Unfortunately, for group F, statistical analysis was not carried out due to the small sample size (n. 3).

The results obtained comparing the *Legionella* community concentrations for each group are summarized in Table 2.

**Table 2:** Comparison of distribution of *Legionella* species within the groups

Groups	*Legionella* comparison	Mann–Whitney test *p* value	*Legionella* comparison	Mann–Whitney test *p* value
A	*Lp*1 ≠ *L. anisa*	1.22 × 10^−7^*	*Lp*1 > *L. anisa*	6.09 × 10^−8^*
	*Lp*1 ≠ *L. rubrilucens*	2.20 × 10^−16^*	*Lp*1 > *L. rubrilucens*	2.20 × 10^−16^*
	*L. anisa* ≠ *L. rubrilucens*	1.04 × 10^−9^*	*L. anisa* > *L. rubrilucens*	5.21 × 10^−10^*
C	*L. anisa* ≠ *L. rubrilucens*	4.76 × 10^−9^*	*L. anisa* > *L. rubrilucens*	2.38 × 10^−9^*
G	*Lp*1 ≠ *L. anisa*	0.028*	*Lp*1 > *L. anisa*	0.014*
H	*Lp*1 ≠ *L. rubrilucens*	0.66	*/*	/
I	*Lp*1 ≠ *L. anisa*	0.47	*/*	/
	*Lp*1 ≠ *L. rubrilucens*	0.57	*/*	/
	*L. anisa* ≠ *L. rubrilucens*	0.80	*/*	/

*p ≤ 0.05

In the hospital water distribution system, in groups A, C, and G, the *Lp*1 population showed the highest concentration, with significant differences with respect to *L. anisa* and *L. rubrilucens.* The lowest contamination was found for *L. rubrilucens.*

To correlate the *Legionella* community and the physical–chemical parameters, such as temperature and disinfectant residues, Spearman’s rho correlation tests were used for all possible pairwise combinations (*Lp*1, *L. anisa*, and *L. rubrilucens*; temperature; and disinfectant residues).

In Table 3, we show the general correlation found between all 191 positive samples and the two main groups represented by *L. pneumophila* and n-*pL*.

**Table 3:** Correlation between *Legionella*, temperature, and disinfectant residues between the three macro-groups (A, B and C)

Comparison (Spearman’s rho correlation test)		Groups		
		A	B	C
		Groups overview		
		All 191 samples	Only *Lp*1	Only n-*pL*
*Lp*1 vs	*L. anisa*	−0.10		
	*L. rubrilucens*	0.09		
	temperature	−0.23**	−0.14	
	H_2_O_2_/Ag^+^ residues	−0.11	0.11	
*L. anisa* vs	*L. rubrilucens*	0.08		−0.25
	temperature	−0.28***		−0.29
	H_2_O_2_/Ag^+^ residues	0.15*		0.14
*L. rubrilucens* vs	temperature	−0.10		0.12
	H_2_O_2_/Ag^+^ residues	−0.11		−0.41**

*p ≤ 0.05, **p ≤ 0.01, ***p ≤ 0.001

In Table 4, it is possible to assess the correlation within each group represented by samples contaminated by only one species (*Lp*1 or *L. anisa* or *L. rubrilucens*) and their interaction when they are present in multiple combinations in the same sample.

**Table 4:** Correlation between *Legionella*, temperature, and disinfectant residues among the groups

Comparison (Spearman’s rho correlation test)		Groups						
		B	D	E	F	G	H	I
		Groups overview						
		Only *Lp*1	Only *L. anisa*	Only *L. rubrilucens*	*L. anisa* and *L. rubrilucens* simultaneously	*Lp*1 and *L. anisa* simultaneously	*Lp*1 and *L. rubrilucens* simultaneously	*Lp*1, *L. anisa*, and *L. rubrilucens* simultaneously
*Lp*1 vs	*L. anisa*					0.33*		0.41
	*L. rubrilucens*						0.60*	0.23
	Temperature	−0.14				−0.33*	−0.42	−0.20
	H_2_O_2_/Ag^+^ residues	0.11				−0.09	−0.86***	0.36
*L. anisa* vs	*L. rubrilucens*				/			−0.02
	Temperature		−0.14		/	−0.30*		−0.34
	H_2_O_2_/Ag^+^ residues		0.12		/	−0.25		0.20
*L. rubrilucens* vs	Temperature			0.02	/		−0.36	−0.05
	H_2_O_2_/Ag^+^ residues			−0.36	/		−0.53*	−0.07

*p ≤ 0.05, **p ≤ 0.01, ***p ≤ 0.001

In group A, regarding the *Legionella* community, we found a negative correlation (−0.10) between *Lp*1 and *L. anisa*, without statistically significance differences. Increasing *Lp*1 led to a decrease in *L. anisa*, or vice versa. By contrast, the correlation within *Lp*1 and *L. rubrilucens* was positive, with a cohabitation of the two species in the same samples, despite the absence of statistically significant differences.

Regarding group C, the correlation between *L. anisa* and *L. rubrilucens* was negative (−0.25), without statistically significant differences. Concerning group G, the correlation between *Lp*1 and *L. anisa* was positive, with statistically significant differences (0.33, *p* = 0.02). Moreover, in group H, the correlation between *Lp*1 and *L. rubrilucens* was positive and statistically significant (0.60, *p* = 0.02). In conclusion, in group I, the correlation between *Lp*1 and *L. anisa*, as well as *Lp*1 and *L. rubrilucens*, was positive (0.41 and 0.23, respectively), despite no statistically significant differences. Instead, *L. anisa* and *L. rubrilucens* showed a non-significant negative correlation (−0.02).

The analysis of the correlation between *Legionella* and the physical–chemical parameters showed interesting results. In group A, a negative correlation was found for all *Legionella* strains (*Lp*1, *L. anisa*, *L. rubrilucens*) and temperature (−0.23, −0.28, and − 0.10, respectively); an increase in temperature led to a decrease in mean *Legionella* concentration, with significant correlations for *Lp*1 (*p* = 1.7 × 10^−3^) and *L. anisa* (*p* = 1.0 × 10^−4^). The analysis of the effect of the disinfectant concentration on the *Legionella* community, in group A, showed a positive significant correlation with *L. anisa* (0.15, *p* = 0.042); increasing the disinfectant dosage led to an increase in the *L. anisa* concentration. On the contrary, for *Lp*1 and *L. rubrilucens*, the data showed a non-significant negative correlation (−0.11, for both species) with the disinfectant concentration; the disinfectant interfered with the *Legionella* concentration.

In group B, a negative correlation was observed (−0.14) between *Lp*1 and temperature, although without significant results. Regarding the effect of disinfectant, *Lp*1 showed a non-significant positive correlation (0.11); disinfectant affected the *Lp*1 concentration.

Group C showed a negative correlation (−0.25) between *L. anisa* and temperature. On the other hand, *L. rubrilucens* maintained its non-significant positive correlation (0.12).

Regarding the effect of disinfectant on the *Legionella* community, a positive correlation (0.14) with *L. anisa* was found, without statistically significant differences. In spite of this, the correlation found with *L. rubrilucens* was a significant negative correlation (−0.41, *p* = 7.3 × 10^−3^); an increased disinfectant dosage caused a decrease in the *L. rubrilucens* concentration.

In group D, the correlation between *L. anisa* and temperature showed non-significant negative result (−0.14). Regarding the effect of disinfectant, *L. anisa* showed a non-significant positive correlation (0.05). Temperature could interfere with *L. anisa* growth, but disinfectant did not impact *L. anisa*.

In group E, the correlation between *L. rubrilucens* and temperature showed a non-significant positive result (0.02). The effect of disinfectant on *L. rubrilucens* showed a non-significant negative correlation (−0.36).

The correlation found in group G between *Lp*1 and *L. anisa* and temperature showed a significant negative value (−0.33, *p* = 0.02 and − 0.30, *p* = 0.04, respectively). Regarding the effect of disinfectant on *Legionella spp.*, both *Lp*1 and *L. anisa* showed a non-significant negative correlation (−0.09 and − 0.25, respectively).

Regarding group H, the correlation between *Lp*1 and *L. rubrilucens* with temperature was a non-significant negative correlation (−0.42 and − 0.36, respectively). The same trend was observed for disinfectant concentration, but with significant result both for *Lp*1 (−0.86, *p* = 1.0 × 10^−5^) and *L. rubrilucens* (−0.53, *p* = 0.04).

The results obtained in group I highlighted a non-significant negative correlation between *Lp*1, *L. anisa*, and *L. rubrilucens* and temperature (−0.20, −0.34, and − 0.05, respectively). Regarding the effect of disinfectant on *Legionella*, a non-significant positive correlation with *Lp*1 and *L. anisa* was found (0.36 and 0.20, respectively). The disinfectant residues and *L. rubrilucens* showed a negative correlation (−0.07).

## Discussion

Our research aimed to study the ecology of the *Legionella* community in a hospital distribution system, using data regarding *Legionella* contamination recorded over 7 years of environmental surveillance. The data presented in our previous studies [27, 28] regarding *Legionella* contamination, its modulation over the year, and the variety of populations found in the hospital suggested the need for a study of correlations among the *Legionella* community and the interaction of *Legionella* species with physical and chemical parameters.

In 7 years of *Legionella* surveillance, the hospital was colonized by *Lp*1 and two species of n-*pL*: *L. anisa* and *L. rubrilucens*, with changes in the level of contamination over time.

Starting from general contamination data, the strain with a higher frequency of isolation was *Lp*1 (78.53%), followed by *L. anisa* (54.45%) and *L. rubrilucens* (21.99%). In some cases, they were present at the same time in the same sample. Understanding the relationship of the *Legionella* community and its interactions with physical and chemical parameters could contribute to explaining the dynamic of contamination in a complex water distribution system, such as the hospital environment.

Although several studies have reported the presence of different species of *Legionella* in the same samples during environmental surveillance, few have referred to the interaction between the most common, *L. pneumophila*, and less documented species, *L. anisa* and *L. rubrilucens*, in the environment, as well as in clinical samples [26–28, 37, 38].

As widely documented by epidemiological data, *Lp*1 is the major causative agent of legionellosis [3]; *L. anisa* is often linked to single cases or epidemic events [39, 40]. The knowledge about etiology, as well as pathogenicity, of *L. rubrilucens* is less than that of *Lp*1 and *L. anisa,* with few clinical cases reported [38, 41]. Few studies have reported human coinfection by *L. pneumophila* with *L. anisa* or *L. pneumophila* with *L. rubrilucens* [38, 42]. Poor knowledge is also reported in environments where the bacteria community is influenced by other bacteria, water, and pipeline features. For this reason, the study of interactions among these species is necessary.

Based on our results, the *Legionella* community had different outcomes. The analysis of correlations among all samples, as presented in group A, showed a negative correlation between *Lp*1 and *L. anisa* that was not observed in other groups. The increase in one of the two species leads to a decrease in the other one, displaying an antagonistic relationship, as previously described by Van Der Mee-Marquet et al. [26]. On the other hand, *Lp*1 was not affected by the presence of other species, such as *L. rubrilucens*, as these two *Legionella* species seem to live in the same ecological niche without interfering with one another. Instead, a positive correlation between *L. anisa* and *L. rubrilucens* was observed only in group A; therefore, the two species may be cohabitants of the same environment in a symbiotic relationship. In other groups, a negative correlation was evident, with a score that was low to intermediate.

Regarding the correlation of *Legionella* with the physical parameters measured, our data confirmed the positive effect of temperature on *Legionella* control [10, 11, 43]. The maximum mean temperature recorded in the hospital outlets was 54.5 °C (48.83 ± 2.59 °C); this is able to control both *Lp*1 and *L. anisa.* These species suffered with increasing temperature, as demonstrated by the negative correlations found in all groups. The results displayed significant differences in 2/5 groups for *Lp*1 (*p*_*GROUP A*_ = 0.002 and *p*_*GROUP* G_ = 0.02), and in 2/5 groups for *L. anisa* (*p*_GROUP A_ = 1.0 × 10^−4^ and *p*_GROUP G_ = 0.039). *L. rubrilucens* showed a little/low correlation with temperature, and only group H showed a medium degree coefficient correlation (−0.36) that could have been associated with a resistance to high temperature. This observation, together with the low concentration of *L. rubrilucens* found in the samples, could explain the thermo-tolerant effect of temperature among *Legionella*, as demonstrated for *L. pneumophila* which are able to change into viable, but non-cultivable, forms [44]. Moreover, resistance to temperature seems to be acquired by members of the genus *Legionella* within species and at the genus level by inter- and intra-species, through spontaneous mutations or horizontal gene transfer [45–47].

Given that the eradication of *Legionella* is difficult, especially when water system colonization occurs, the simultaneous action of high temperature and disinfection treatment is a valid strategy to control *Legionella* colonization [17].

As reported in our previous study [27], H_2_O_2_/Ag^+^ presents economic and operative advantages as disinfection treatment, with good performance in continuous hospital disinfection treatment. Changes occurred, during the study, in the concentration and *Legionella* species isolated, suggesting a possible role or interaction between *Legionella* and the disinfectant that could only be investigated by studying their correlation.

*Lp*1 was generally affected by H_2_O_2_/Ag^+^, with statistically significant results (*p*_GROUP H_ = 1.0 × 10^−5^ and a high degree of coefficient correlation, −0.86). *L. anisa* showed a probable resistance to H_2_O_2_/Ag^+^ treatment, with significant results found in group A (*p*_GROUP A_ = 0.042). *L. rubrilucens* was always sensitive to H_2_O_2_/Ag^+^ treatment, with significant results (*p*_GROUP C_ = 0.0073 and *p*_GROUP H_ = 0.042). These varied results for different *Legionella* species could be explained considering that the continuous dosage of disinfectant recorded in the hospital had a concentration of 11.58 ± 4.99 mg/L, which was probably too low to achieve good control of the *Legionella* community. Casini et al. [19], for example, suggest that the dosage to control *Legionella* contamination in the hospital should be 25 mg/L. Moreover, the presence of catalase or peroxidase in *Legionella* spp. can increase tolerance to H_2_O_2_ at low concentrations [48, 49]. An increase in dosage could improve the action of H_2_O_2_/Ag^+^ on all *Legionella* communities, especially *L. pneumophila* and *L. anisa*, which seem be more resistant to treatment with respect to *L. rubrilucens*. We must consider that, when the treatment is performed over time, as in the hospital investigated in this study, it is possible to find persistence and an increase in the concentration of these species. Furthermore, the different and selective activity of H_2_O_2_/Ag^+^ on *Legionella* species could be investigated and explained by the presence of superoxide dismutase activity (SOD), used by Gram-negative bacteria, as well as *Legionella*. For example, in *L. pneumophila*, periplasmic SODs of copper and zinc (CuZnSOD) contribute to survival during the stationary phase of growth, and can enhance bacteria pathogenicity [50]. Consequently, the existence of CuZnSOD in *L. pneumophila* could be one of the reasons for its survival despite the disinfection treatment. Unfortunately, there is no evidence regarding the presence or activity of this system in other *Legionella* species.

A study of the “in vitro” response to the H_2_O_2_/Ag^+^ compound used in this hospital could contribute to clarifying the different responses by the species analyzed.

This study, as far as we know, is the first to evaluate the interaction between three different *Legionella* species, and their interaction with temperature and disinfection treatment, in the same water distribution system. This study demonstrated how the presence of one species or more, in the same environment, could contribute to enhancing the growth of species concentration, resistance to disinfectant, or sensitivity to temperature increases.

At the same time, this study raises a new consideration regarding the complexity of the *Legionella* community, which needs to be investigated further, including giving more attention to lesser known *Legionella* species. In conclusion, the aquatic environment is a natural reservoir for several bacteria (pathogenic and non-pathogenic) that live in symbiosis or in competition.

Understanding the interaction between the *Legionella* community and the aquatic environment is valuable not only to obtain and program the right strategies to control these bacteria (e.g., temperature values or disinfectant concentrations), but also to support us to better understand the changes in *Legionella* communities that occur during extensive disinfection treatment. The surveillance of Legionellosis must consider not only the bacteria concentration, but also the identification of bacterial communities that are influenced by interactions with the environment, water characteristics, and the choice of pipeline system.

## Data Availability

Not applicable.
